# Protocol for automated quantification of neuronal cells using the Agilent BioTek Cytation system and Gen5 neurite outgrowth module

**DOI:** 10.1016/j.xpro.2026.104485

**Published:** 2026-04-01

**Authors:** Aline Broeglin, Olivier Buetzberger, Rebecca Mongeon, Aurélien Riou, Anne Eckert, Amandine Grimm

**Affiliations:** 1 Research Cluster, Molecular & Cognitive Neuroscience, Department of Biomedicine, University of Basel, 4001 Basel, Switzerland; 2 Neurobiology Laboratory for Brain Aging and Mental Health, University Psychiatric Clinics (UPK), 4002 Basel, Switzerland; 3 Agilent Technologies, EPFL Innovation Park, 1015 Lausanne, Switzerland; 4 Agilent Technologies, Winooski, VT 05404, USA

**Keywords:** Cell Biology, High Throughput Screening, Microscopy, Neuroscience

## Abstract

Drugs or pathological proteins can influence neuronal plasticity. Here, we provide a protocol to quantify neurite outgrowth parameters in differentiated SH-SY5Y neuroblastoma cells using the Agilent BioTek Gen5 neurite outgrowth module. The protocol includes steps for plating cells, inducing differentiation, performing immunostaining, collecting images automatically, and analyzing neurite morphology. This assay enables the evaluation of neuronal plasticity and assesses the effects of drugs or pathological proteins. It can be adapted to other neuronal models and scaled for high-throughput screening.

## Highlights

• Cell culture and sample preparation procedures for neurite outgrowth analysis
• Automated microplate imaging and analysis setup on the Agilent BioTek platform
• Instructions to optimize settings for accurate neurite outgrowth quantification

## Abstract (editor highlights)

Publisher’s note: Undertaking any experimental protocol requires adherence to local institutional guidelines for laboratory safety and ethics.

## Graphical abstract

**Figure:**
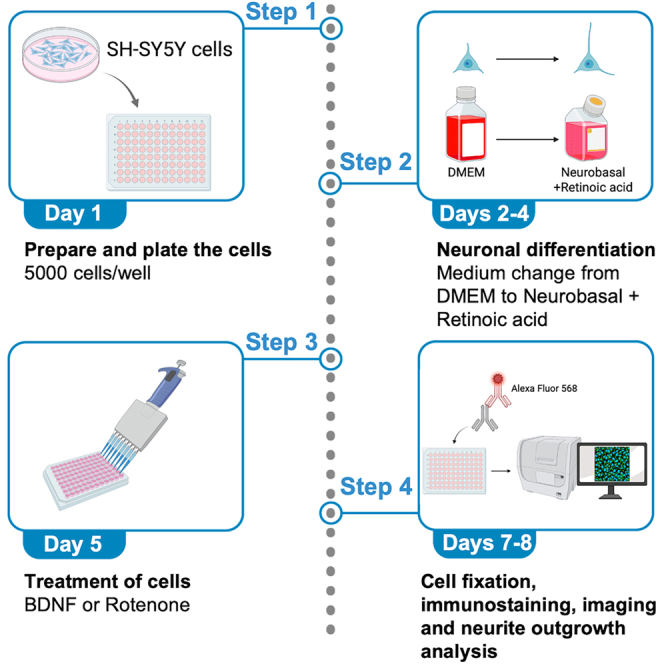

## Teaser

Drugs or pathological proteins can influence neuronal plasticity. Here, we provide a protocol to quantify neurite outgrowth parameters in differentiated SH-SY5Y neuroblastoma cells using the Agilent BioTek Gen5 neurite outgrowth module. The protocol includes steps for plating cells, inducing differentiation, performing immunostaining, automated image collection, and analyzing neurite morphology. This assay enables the evaluation of neuronal plasticity and assesses the effects of drugs or pathological proteins. It can be adapted to other neuronal models and scaled for high-throughput screening.

## Before you begin

This protocol focuses on assessing neurite outgrowth capacity in differentiated SH-SY5Y cells treated with brain-derived neurotrophic factor (BDNF), rotenone, or engineered to express Alzheimer’s disease-linked pathological proteins such as amyloid precursor protein (APP) and mutant tau (P301L). These treatments and cell models were selected to demonstrate the sensitivity and accuracy of the Agilent BioTek Gen5 software Neurite Outgrowth module in detecting subtle alterations in neurite outgrowth parameters induced by pharmacological or genetic perturbations.

The native human neuroblastoma cell line SH-SY5Y comes from ATCC (CRL-2266).

The P301L cells are SH-SY5Y cells stably overexpressing the P301Ltau mutation in the microtubule-associated protein Tau (MAPT) gene. Cells were kindly provided by Prof. Jürgen Götz Laboratory (Queensland Brain Institute, University of Queensland, Brisbane, Australia), which generated the cell line using lentiviral gene transfer.^1^ Cell clones stably expressing the full-length human hTau40 bearing the P301L mutation and a green fluorescent protein (GFP) tag were selected by adding 4.5 μg/mL of blasticidin to the culture medium. We previously showed that these cells exhibit abnormal tau hyperphosphorylation and mitochondrial impairments.^2^^,^^3^

The APP cells are SH-SY5Y cells stably overexpressing the entire coding region of human wild-type APP (APP695). The cell line was generated using Lipofectamine Plus mixture.^4^ Cell clones stably expressing APP were maintained in medium containing 300 μg/mL hygromycin to ensure stable vector expression. We previously showed that APP cells secrete Amyloid-β_1-40_ at levels in the pg/mL range and exhibit mitochondrial impairments.^5^

All the steps described below are performed under sterile conditions until the culture fixation step.

### Innovation

Neuronal morphology is a key indicator of neuronal health and plasticity, highlighting the need for robust and reproducible neurite quantification methods. This protocol provides a rapid, standardized, and flexible workflow for quantitative neurite analysis using the Agilent Gen5 Neurite Outgrowth Module, applicable to a wide range of neuronal models.

### Preparation of cell culture media

**Timing: 10 min**

1. Prepare the SH-SY5Y culture growth medium.
  a. Add 10% of fetal bovine serum, 5% of horse serum, 1% of penicillin-streptomycin and 1% GlutaMax in Dulbecco’s Modified Eagle Medium – high glucose (DMEM).
  b. Mix the complete growth medium well.
  c. Store at +4°C until use.

***Note:*** The growth medium can be stored at 4°C for up to 1 month.

***Note:*** The APP and P301L cells were maintained in the growth medium with the antibiotic of selection, hygromycin (300 μg/mL) or blasticidin (4,5 μg/mL), respectively, and kept under steady selection pressure. The antibiotics are directly added to the petri dishes with the cells.

### Cell culture

**Timing: 30 min**

2. Cell passaging.
  a. Check the confluency of cells under the microscope.
    ***Note:*** Cells are grown in 10 cm^2^ culture dishes containing 10 mL of growth medium and split at approximately 80% confluency.
  b. Remove the medium from the dish by aspiration.
  c. Wash once with 10 mL of 1× PBS (−) for 1 min.
    ***Note:*** The PBS (−) does not contain calcium and magnesium.
  d. Add 1.5 mL Accutase to the dish to cover the whole surface.
  e. Aspirate the Accutase from the dish.
  f. Incubate the dish for approximately 5 min at 20°C (lid closed to avoid cells’ drying).
    ***Note:*** SH-SY5Y are adherent cells. Check the cells under a microscope to confirm they are detached before proceeding to the next steps.
  g. Resuspend the cells in growth medium (see “preparation of cell culture media”).
    ***Note:*** The volume of growth medium depends on cell confluency. At ∼80% confluency, cells are typically resuspended in 4–5 mL of medium.
  h. Gently transfer the cell suspension to a 15 mL conical tube by pipetting.
  i. Transfer the cells to a 15 mL tube containing the growth medium. (see “preparation of cell culture media”).
  j. Prepare two new 10 cm^2^ dishes with 10 mL of fresh growth medium.
  k. Distribute a fraction of recovered cells into these two new dishes.
    ***Note:*** Prepare one dish for the experiments and one for maintaining cells in culture for future experiments.
  l. Gently distribute the cells using a figure- 8 movement to assure homogeneous plating.
    ***Note:*** Cells grow better when they are evenly distributed in the dish.
3. Antibiotic selection.
  a. Add 4.5 μg/mL of blasticidin to the P301L cell dish.
  b. Add 300 μg/mL of hygromycin to the APP cell dish.

***Note:*** To maintain steady selection pressure, blasticidin and hygromycin are added to dishes containing P301L- or APP-overexpressing cells.

4. Maintain cells in an incubator at 37°C and 5% CO_2_.

### Preparation of 4% formaldehyde solution for cell fixation

**Timing: 1 h**

***Note:*** Paraformaldehyde (PFA) is toxic; all the steps must be performed with gloves under a ventilated hood. Safety glasses are also recommended. Commercially available 4% formaldehyde solutions can be used as an alternative.

5. This protocol generates 1L of 4% formaldehyde cell fixation solution.
  a. Heat 800 mL of 1× PBS (−) to 60°C.
  b. Add 40 g PFA powder to the heated 1× PBS.
    ***Note:*** To maintain the temperature and help dissolve the powder, it is recommended to use a hot plate with a magnetic stirrer.
  c. Add NaOH dropwise until the PFA powder is fully dissolved.
  d. Adjust the final volume to 1L with 1× PBS (−).
  e. Adjust the pH to 6.9 with HCl solution.
6. Filtration and storage.
  a. Filter the formaldehyde solution with a 0.2 μm mesh size filter.
    ***Note:*** Formaldehyde solution must be cooled for this step.
  b. Store the solution in a glass bottle at +4°C.
    ***Note:*** The 4% formaldehyde solution can be stored up to 1 month at 4°C. The solution can also be aliquoted and frozen at −20°C.

## Key resources table

**Table:** 

REAGENT or RESOURCE	SOURCE	IDENTIFIER
**Antibodies**		

Primary antibody anti-βIII tubulin mouse	R&D	MAB 1195; RRID: AB_357520
Secondary antibody goat anti-mouse coupled Alexa Fluor 568	Abcam	175473; RRID: AB_2895153

**Chemicals, peptides, and recombinant proteins**		

Accutase	Innovative Cell Technologies, Inc.	Cat#AT-104
Blasticidin	Invivogen	Cat#Ant-bl
BSA	Sigma-Aldrich	Cat#A9647
B-27	Gibco	Cat#17504001
DAPI	Merck	Cat#10236276001
DMEM	Sigma-Aldrich	Cat#D6429
Fetal bovine serum	Corning	Cat#35-079-CV
GlutaMax	Thermo Scientific	Cat#35050087
Horse serum	BioConcept	Cat#2-05F00-1
Hygromycin	Roth	Cat#CP12.2
Laminin	Sigma-Aldrich	Cat#11243117001
Neurobasal	Gibco	Cat#21103-049
PBS(+)	Sigma-Aldrich	Cat#D8662
PBS(−) 10×	Dominique Dutscher	Cat#MS019N1008
PFA	Sigma-Aldrich	Cat#P6148
Penicillin/Streptomycin	Bioconcept	Cat#4-01F00-5
Retinoic acid	Sigma-Aldrich	Cat#R2625
Triton	Sigma	Cat#9002-95-1

**Deposited data**		

Raw data	This paper	Open Science Framework: https://osf.io/26tgc/overview?view_only=1ca624c35de949a78bc3a8c9f8077813

**Experimental models: Cell lines**		

APP transfected SH-SY5Y neuroblastoma cells (Human)	The Eckert laboratory	N/A
P301L-Tau-GFP transfected SH-SY5Y neuroblastoma cells (Human)	The Götz laboratory, Queensland Brain Institute, University of Queensland, Brisbane, Australia	N/A
SH-SY5Y neuroblastoma cells (Human)	ATCC	CRL-2266

**Software and algorithms**		

Agilent BioTek Gen5 Image Prime Software, Version 3.16	Agilent Technologies	N/A
Neurite Outgrowth Module	Agilent Technologies	N/A
Prism version 10.5.0	GraphPad	RRID: SCR_002798; https://www.graphpad.com/features

**Other**		

Culture dishes 10 cm^2^	Sarstedt	Cat#83.3902.300
Parafilm “M”	Amcor	Cat#PM-996
Black 96 well plates	Agilent Technologies	Cat#204626-100
Agilent BioTek Cytation Cell Imaging Multi-Mode Reader	Agilent Technologies	Cat#CYT5MPW
20× Olympus Plan Fluorite Objective	Agilent Technologies	Cat#1220517
Laser Autofocus Cube	Agilent Technologies	Cat#1225010
DAPI LED and Filter Cube	Agilent Technologies	Cat#1225007, Cat#1225100
RFP LED and Filter Cube	Agilent Technologies	Cat#1225003, Cat#1225103
CMOS Camera	Sony	Cat#IMX 264

## Materials and equipment

**Table:** Growth Medium

Reagent	Final concentration	Amount
Dulbecco’s Modified Eagle Medium (DMEM)	N/A	500 mL
Fetal Bovine Serum	10%	50 mL
Horse Serum	5%	25 mL
GlutaMax	1%	5 mL
Penicillin-Streptomycin	1%	5 mL

Storage conditions: Store the Dulbecco’s Modified Eagle Medium at +4°C. Aliquot and store the other compounds at −20°C for up to 12 months. Store the growth medium at +4°C for up to 4 weeks.

**Table:** Differentiation medium

Reagent	Final concentration	Amount
Neurobasal Medium	N/A	15 mL
B-27	2%	300 μL
Penicillin-Streptomycin	1%	150 μL
GlutaMax	1%	150 μL
Retinoic Acid	10 μM [Stock= 110 mM]	1.2 μL

Storage conditions: Store the neurobasal medium at +4°C. Aliquot and store the retinoic acid at −20°C for up to 6 months. Aliquot and store all the other compounds at −20°C for up to 12 months. Use the differentiation medium fresh, immediately after preparation.

## Step-by-step method details

### Preparation of the plate coating and cell plating

**Timing: 1 h 30 min**

This section of the protocol focuses on the coating preparation for the plates containing the 3 cell lines (native SH-SY5Y, APP, and P301L cells). Although SH-SY5Y cells are adherent, this coating step contributes to the differentiation process. In parallel with coating preparation, cell counting is performed to ensure uniform cell distribution in each well. The cells used for the experiments were cultured as described above and were prepared for the experiments as described below. All conditions are prepared in duplicate or triplicate for each experiment.

1. Coating of the black-walled 96-well microplates with laminin.
  **CRITICAL:** Black-walled microplates with clear bottoms are recommended for fluorescence microscopy to minimize light reflection and reduce the fluorescence background.
  a. Prepare a 10 μg/mL solution of laminin in PBS (+) from the 0.5 mg/mL stock solution.
    ***Note:*** The PBS (+) is supplemented with calcium and magnesium.
  b. Incubate the plate at 37°C for 1h.
2. Cell counting
  a. Detach the cells from the dish as described in the « cell culture »section.
  b. Dilute the cells 1:10 in Trypan Blue.
  c. Count the cells with a hemocytometer under the microscope.
    ***Note:*** We used a Neubauer hemocytometer. The formula to determine the cell count is: $\left(\frac{Cell\;number\;chamber\;1+Cell\;number\;chamber\;2}{2}\right)\times Dilution\;factor\times 10^{4}$. Other counting procedures can be used.
  d. Dilute the cells in the growth medium to a final concentration of 5,000 cells/well.
    ***Note:*** Depending on the cell line used, it is recommended to seed between 2,500 and 10,000 cells per well to avoid cell overlap. (See the troubleshooting section, problem 1).
3. Cell plating
  a. Seed 100 μL/well of cells suspension in the coated 96-well microplates.
    ***Note:*** Do not seed cells into the outer wells to prevent variability caused by medium evaporation.
  b. Add 200 μL of 1× PBS (−) in the outer wells.
    ***Note:*** This step reduces the evaporation of the growth medium.
  c. Incubate for 1h at 20°C.
    ***Note:*** This step is recommended to improve cell adherence to the coating. See also the troubleshooting section, problem 2.
  d. Incubate the plate at 37°C and 5% CO_2_.

### Cell differentiation and treatment with BDNF or rotenone

**Timing: 5 days**

This section describes the differentiation protocol used to induce neurite outgrowth in SH-SY5Y cells, followed by treatments with brain-derived neurotrophic factor (BDNF) or rotenone.

BDNF promotes neurite outgrowth, whereas rotenone inhibits this process.^6^^,^^7^

The goal of this experiment is to assess the impact of both compounds on the neurite outgrowth phenotype across our neuronal models.

4. Cell differentiation
  a. Prepare the differentiation medium:
    Neurobasal + 1% Penicillin-streptomycin + 1% GlutaMax + 2% B-27 + 10 μM Retinoic acid
    ***Note:*** Retinoic acid is dissolved in DMSO and stored at −20°C in aliquots to limit freeze–thaw cycles (Stock concentration: 110 mM). This compound is light-sensitive; this step must be performed under dim light conditions. The differentiation medium is used directly after preparation.
  b. Aspirate the growth medium from the wells.
  c. Wash once with 100 μL/well of 1× PBS (−) for 1 min.
  d. Add 100 μL/well of the differentiation medium.
  e. Incubate the plate at 37°C and 5% CO_2_ for 72h.
5. Treatment with BDNF or rotenone.
  a. Dilute BDNF and rotenone in Neurobasal + 1% Penicillin-streptomycin + 1% GlutaMax to the final concentrations:
    i. BDNF: 10, 50, and 100 ng/mL.
    ii. Rotenone: 0.05, 0.1, and 0.5 μM.
      ***Note:*** Aliquots of BDNF and rotenone are stored at −20°C. Avoid repeated freeze-thaw cycles.
  b. Treat the differentiated cells by adding 10 μL/well of compound.
  c. Incubate the plate at 37°C and 5% CO_2_ for 48h.

### Immunostaining of βIII-tubulin for neurite outgrowth analysis

**Timing: 2 h 30 min**

This section outlines the immunostaining for βIII-tubulin to visualize neurites for image-based outgrowth analysis. Negative controls are essential to validate immunostaining specificity and background levels; therefore, samples processed i) without primary antibody, ii) without secondary antibody, or iii) without any antibody should be included to assess non-specific binding and cellular autofluorescence. DAPI is a nuclear counterstain used to identify the nucleus and optimize neuronal soma identification with the Gen5 software Neurite Outgrowth module.

6. Cell fixation.
  a. Warm the 4% formaldehyde solution to 37°C in a water bath.
  b. Aspirate the differentiation medium.
  c. Add 50 μL/well of pre-warmed 4% PFA solution.
  d. Incubate for 10 min at 20°C.
  e. Wash three times with 100 μL/well of 1× PBS (−) for 1 min.

***Note:*** Formaldehyde waste must be collected in a dedicated chemical waste container and disposed of in accordance with institutional safety regulations.

***Note:*** The following steps do not require sterile conditions.

7. Cell permeabilization and blocking of nonspecific binding sites.
  a. Prepare 0.15% Triton X-100 in 1× PBS (−).
  b. Remove the PBS in the wells.
  c. Add 50 μL/well of the 0.15% Triton X-100 solution.
  d. Incubate for 15 min at 20°C.
    ***Note:*** This step reveals the epitope to the primary antibody.
  e. Prepare 2% BSA solution in PBS (−) for blocking nonspecific binding sites.
    ***Note:*** 1% = 10 mg/mL.
  f. Wash the wells three times with 100 μL/well of 1× PBS (−) for 1 min.
  g. Add 50 μL/well of the 2% BSA solution.
  h. Incubate for 1h at 20°C.
    ***Note:*** See the troubleshooting section, problem 3.
8. Primary antibody incubation.
  a. Dilute the anti-βIII-tubulin antibody 1:1000 in 1% BSA solution.
    ***Note:*** Reference of primary antibody: R&D MAB 1195 anti-βIII tubulin, Mouse.
  b. Aspirate the blocking buffer.
  c. Add 50 μL/well of the primary antibody solution.
  d. Incubate overnight at +4°C.
    ***Note:*** Seal the plates with Parafilm to prevent evaporation.
9. Secondary antibody and nuclear staining.
  a. Wash three times with 100 μL/well of 1× PBS (−) for 1 min.
  b. Dilute 1:1000 the anti-mouse antibody in 1% BSA solution.
    ***Note:*** Reference of secondary antibody: Abcam 175473, goat anti-mouse coupled with Alexa 568.
  c. Add 50 μL/well of the secondary antibody solution.
  d. Incubation for 1h at 20°C in the dark.
  e. Wash three times with 100 μL/well of 1× PBS (−) for 1 min.
  f. Dilute DAPI 1:1000 in 1× PBS (−).
  g. Add 50 μL/well of the DAPI solution.
  h. Incubate for 5 min at 20°C in the dark.
  i. Wash three times with 100 μL/well of 1× PBS (−) for 1 min.
    ***Note:*** See the troubleshooting section, problem 4.
  j. Seal the plate with Parafilm.
  k. Store the plate at +4°C, protected from light.
    ***Note:*** Plates can be stored at +4°C for 2–4 weeks.

### Image acquisition with the Agilent BioTek Cytation instrument and analysis using the Gen5 software neurite outgrowth module

**Timing: 2 h**

This section describes the image acquisition on the Agilent BioTek Cytation instrument and analysis procedure for neurite outgrowth quantification using the Gen5 software Neurite Outgrowth module. Similar results can be expected from additional Agilent BioTek automated imaging instrumentation. The parameters below are optimized for the cellular models used in this study and should be adjusted for each specific model.

10. Capture a reference image with the DAPI and RFP channels.
  a. Open the Gen5 software.
  b. Click “Manual Mode”.
  c. Enter the parameters corresponding to your experimental conditions:
    i. Objective, Filter set, Microplate format, Vessel type.
  d. Select a reference well.
  e. Select the DAPI channel (nuclear channel, Excitation/Emission: 377/447 nm).
  f. Click “Find image”.
    ***Note:*** The Agilent BioTek automated cell imagers perform autofocus and autoexposure adjustments until a clear image is obtained.
  g. Adjust the image acquisition settings.
    i. Illumination: 10.
    ii. Integration time: 5.
    iii. Gain: 14.
      ***Note:*** Image acquisition settings may need to be adjusted between assays based on staining efficiency and sample properties.
  h. Identify the most representative field of your sample.
  i. Click “Capture”.
    ***Note:*** Image.
  j. Repeat the procedure for the RFP channel (neurite staining channel, Excitation/Emission: 531/593 nm).
    Image acquisition settings.
    i. Illumination: 10.
    ii. Integration time: 34.
    iii. Gain: 24.1.
  k. Click “Image Preprocessing”.
    ***Note:*** Image preprocessing is optional and performs background subtraction using a rolling-ball algorithm, a common processing method used in fluorescence microscopy to better visualize the cellular staining across a broad range of signal-to-noise ratios.
  l. Enter the rolling ball size for each channel:
    i. DAPI: 30 μm.
    ii. RFP: 100 μm.
      ***Note:*** The rolling ball size controls the background smoothing radius and is recommended to be greater than the maximum object size to be detected.
      ***Note:*** Exposure time and camera gain may need to be adjusted during reference image acquisition and preprocessing, depending on the neuronal marker used for immunostaining.
11. Optimize neurite detection settings.
  a. Click “Neurite outgrowth analysis”.
  b. The default parameters provide a good starting point for most cell culture neurite outgrowth analysis. These parameters were further adjusted to optimize neurite and soma detection for this neuronal model (see Table 1).

**Table 1:** Settings for the neurite outgrowth analysis of the SH-SY5Y cells

Neurite Outgrowth Analysis settings	
Soma detection	ßIII-tubulin (RFP channel)
Threshold Slider Level	41
Minimum/Maximum Size	5/100 μm
Soma Closing Size	0 μm
Optimized Soma Using Nuclear Signal	Checked
Rolling Ball Diameter	50

**Nucleus Detection (DAPI Channel)**	

Threshold Slider Level	17
Minimum/Maximum Size	8/100 μm
Minimum Nucleus/Soma Overlap	15%
Maximum Nucleus/Soma Distance	20 μm

**Neurites**	

Threshold Slider Level	−31
Neurite Mask Closing Size	1 μm
Rolling Ball Diameter	5 μm
Only Keep Neurites Connected to a Soma	Checked
Discard Short Ending Branches	10 μm

***Note:*** It is recommended to optimize nucleus and soma detection first, followed by neurite mask optimization.

***Note:*** These settings are optimized for SH-SY5Y cells and should be adjusted for other cell types, particularly nucleus/soma size and rolling ball diameter. Examples of settings for iPSC-derived and primary neuron culture models can be found on the related Application Note.^8^

12. Create the Gen5 experiment.
  a. Click “Create experiment from an image set” button in Gen5 software to import all the imaging and analysis settings (Part 4, Steps 1 and 2) into a new experiment for automated whole-plate imaging.
    ***Note:*** Image resolution is set to 1992 × 1992 pixels.
  b. Define the plate layout.
  c. Set the desired automated focus method.
    ***Note:*** The Laser autofocus option increases acquisition speed and focus reproducibility while reducing the risk of photobleaching.
  d. Images will be collected in the center of the well by default. To change the imaging location, use the “Beacons” feature to set fixed imaging regions of interest.
    ***Note:*** In this setup, five beacons were positioned in the central area and four at the periphery of each well to provide representative coverage of neurite outgrowth across the entire well. It is recommended to set at least 3–4 beacons per well. To apply the same beacon locations to all wells, select the corresponding option.
  e. Click “Run” to automatically collect images and perform neurite outgrowth image analysis.
  f. Neurite outgrowth analysis results are visualized in Gen5 software and can be exported for further evaluation.
  g. Generate summary figures and statistical analysis across datasets.
    ***Note:*** Figures and statistical analyses were performed using GraphPad Prism software.

## Expected outcomes

In this study, two independent experiments were performed. For each condition, 4–6 technical replicates were analyzed. From each well, 5–9 images were acquired and analyzed, with each data point corresponding to a single image. Data were normalized to the control condition (0 ng/mL BDNF, 0 μM rotenone, and Control cells) from a reference experiment. Normalized data from both independent experiments were then pooled, and results are presented as the mean ± standard error of the mean (SEM).

### Impact of BDNF on neurite outgrowth in SH-SY5Y cells

**Figure 1:**
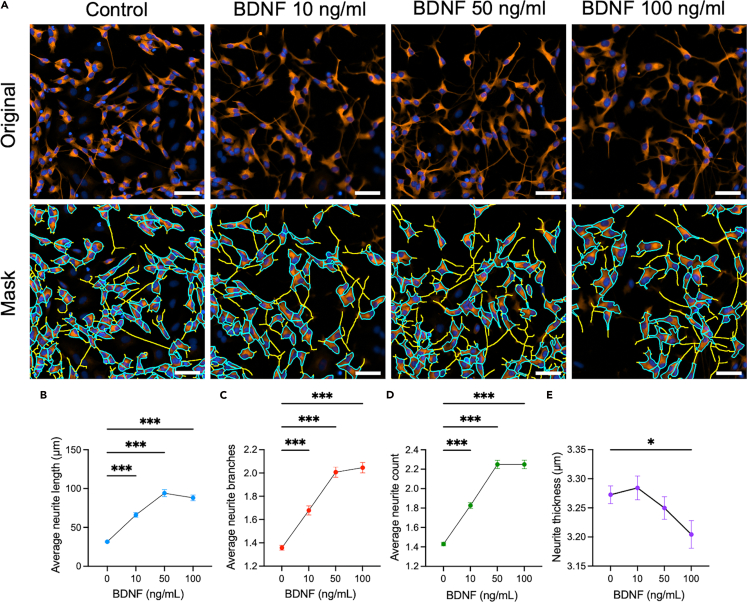
Effect of BDNF treatment on the neurite outgrowth parameters in SH-SY5Y cells Differentiated SH-SY5Y cells were treated with 10 ng/mL, 50 ng/mL, or 100 ng/mL of BDNF for 48h. (A) Cells were stained for ßIII-tubulin (red) and DAPI (blue). All images were captured 48h post-treatment at 20× magnification on the Agilent BioTek Cytation 5 instrument. Image analysis was performed with the Gen5 software Neurite Outgrowth module where the soma mask overlay is displayed in blue and the neurite mask overlay in yellow. (B) Average neurite length, (C) average neurite branches, (D) average neurite count depending on BDNF concentration, and (E) neurite thickness depending on BDNF concentration. Scale bar: 50 μm. Each data set represents N=2 independent experiments with 4-6 replicates per condition and with 5-9 images per well. The points represent the mean ± SEM for each condition. One-way ANOVA and post hoc Dunnett’s multiple comparison tests. ∗*p* < 0.05; ∗∗*p* < 0.01; ∗∗∗*p* < 0.001. BDNF: Brain-derived neurotrophic factor.

Treatment with BDNF, 10 and 50 ng/mL for 48 h (Figure 1A), showed a dose-dependent increase in average neurite length, number of branches, and neurite count compared with the control condition (Figures 1B–1D). In contrast, treatment with 100 ng/mL BDNF led to a significant decrease in neurite thickness relative to the control group (Figure 1E). Overall, these results indicate that BDNF promotes neurite outgrowth in SH-SY5Y cells.

### Impact of rotenone on neurite outgrowth in SH-SY5Y cells

**Figure 2:**
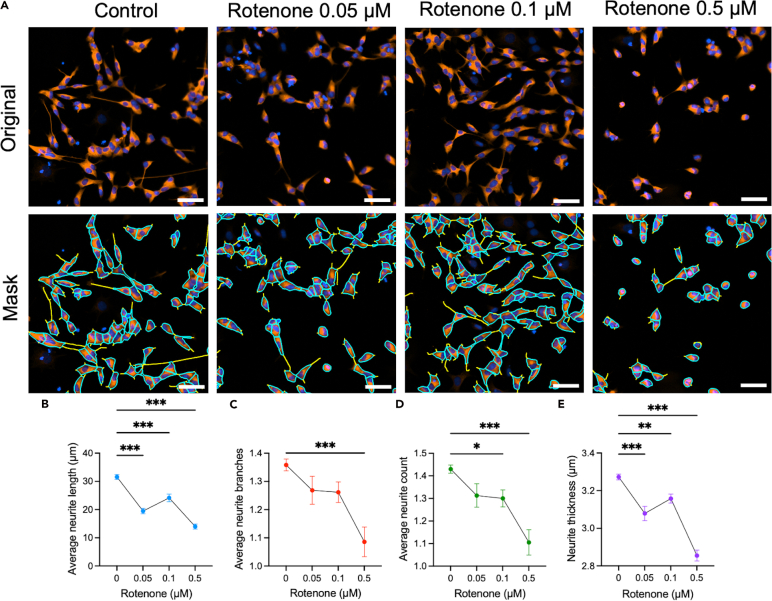
Effect of rotenone treatment on the neurite outgrowth parameters in SH-SY5Y cells Differentiated SH-SY5Y cells were treated with 0.05 μM, 0.1 μM or 0.5 μM of rotenone for 48h. (A) Cells were stained for ß3 tubulin (red color) and DAPI (blue color). All images were captured 48h post-treatment at 20× magnification on the Agilent BioTek Cytation 5 instrument. Image analysis was performed with the Gen5 software Neurite Outgrowth module where the soma mask overlay is displayed in blue and the neurite mask overlay in yellow. (B) Average neurite length, (C) average neurite branches, (D) average neurite count, and (E) neurite thickness depending on rotenone concentration. Scale bar: 50 μm. Each data set represents N=2 independent experiments with 4-6 replicates per condition and with 5-9 pictures per well. The points represent the mean ± SEM for each condition. One-way ANOVA and post hoc Dunnett’s multiple comparison tests. ∗*p* < 0.05; ∗∗*p* < 0.01; ∗∗∗*p* < 0.001.

Treatment with rotenone, 0.05, 0.1, and 0.5 μM for 48 h (Figure 2A), led to a dose-dependent decrease in neurite length and neurite thickness compared with the control condition (Figures 2B–2E). In addition, treatment with 0.5 μM rotenone significantly reduced the number of neurite branches and neurite count compared with the control group (Figures 2C and 2D). These findings demonstrate that rotenone inhibits neurite outgrowth in SH-SY5Y cells.

### Impact of the APP and P301L-Tau mutation overexpression on neurite outgrowth in SH-SY5Y cells

**Figure 3:**
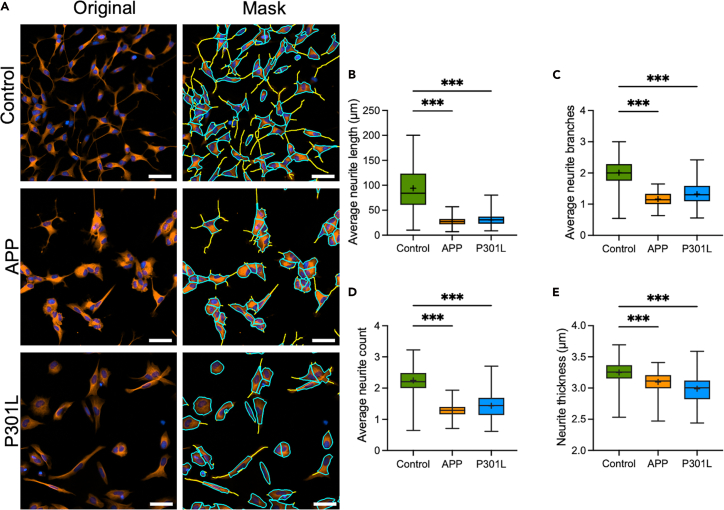
Impact of the APP and P301L mutation on the neurite outgrowth capacity of SH-SY5Y cells The control condition corresponds to the native cells. The APP condition corresponds to the cells stably overexpressing the Amyloid Precursor Protein (APP). The P301L condition corresponds to the cells stably overexpressing the P301L-Tau mutation. The differentiated cells were treated with 50 ng/mL of BDNF for 48h. (A) Cells were stained for ß3 tubulin (red) and DAPI (blue). All images were captured 48h post-treatment at 20× magnification on the Agilent BioTek Cytation 5 instrument. Image analysis was performed with the Gen5 software Neurite Outgrowth module where the soma mask overlay is displayed in blue and the neurite mask overlay in yellow. (B) Average neurite length, (C) average neurite branches, (D) average neurite count, and (E) neurite thickness in Control, APP, and P301L cells. Scale bar: 50 μm. The boxes represent the median (full line) and the mean (“+” symbol); the whiskers represent the minimum and maximum values. Each data set represents N=2 independent experiments with 4-6 replicates per condition. One-way ANOVA and post hoc Dunnett’s multiple comparison tests. ∗*p* < 0.05; ∗∗*p* < 0.01; ∗∗∗*p* < 0.001.

Overexpression of APP and the P301L-Tau mutation both led to a decrease in average neurite length, number of branches, neurite count, and neurite thickness compared with the control condition (Figures 3A–3E). These results indicate that both pathological cellular models exhibit a deficit in neurite outgrowth capacity.

Representative fluorescence images and quantitative analyses of neurite outgrowth were generated using the Agilent BioTek Gen5 software Neurite Outgrowth module and are shown in Figures 1, 2, and 3. These results validate the workflow described here for reliably detecting both stimulatory and inhibitory effects on neuronal morphology.

## Limitations

Automated imaging and analysis with the Agilent BioTek Cytation platform and Gen5 Neurite Outgrowth module provide a fast and standardized method for analyzing fluorescence microscopy images of neuronal cells. The Gen5 Neurite Outgrowth Module is flexible and applicable to diverse cellular models, from neuroblastoma cells to complex neuronal morphologies such as iPSC-derived neurons, enabling quantitative neurite quantification.^8^ However, several limitations should be considered.

Outgrowth analysis depends heavily upon the signal-to-background ratio to accurately detect neurites along their entire length and across diverse cell morphologies. Neuronal morphology analysis benefits from a strong, reliable and relatively continuous signal throughout neurites for proper segmentation. Immunostaining with cytoskeletal markers, such as βIII-tubulin and MAP2, is a recognized approach for accurate and optimal morphology evaluation used broadly across neuronal culture models. Outgrowth analysis with alternative cellular markers should be validated for each model.

In addition, proper segmentation of individual soma in high density cultures and cell clusters can be challenging. The Gen5 software Neurite Outgrowth module can combine the information from nuclear staining and somatic staining to better segment neurons within clusters. Therefore, it is strongly recommended to include a nuclear counterstain (e.g., DAPI) for neuronal segmentation with immunolabeled culture samples to improve soma segmentation and result accuracy.

To automatically detect neurites and soma consistently across wells, the Gen5 Neurite Outgrowth module uses an automated thresholding approach that can account for varying sample intensity across images due to technical or experimental variables. However, if the fluorescence intensity is highly variable within an individual image, regions with very low signal may sometimes result in under-segmentation errors. As with most image-based analysis, the accuracy of the segmentation and results for neurite outgrowth depends upon the quality and consistency of sample staining.

Furthermore, the Gen5 Neurite Outgrowth analysis module provides quantitative neurite parameters but does not inherently distinguish between effects occurring in axonal or dendritic compartments when using a general neurite marker, such as βIII-tubulin. For compartment-specific neurite outgrowth analysis with Gen5 Neurite Outgrowth module, cultures should be labeled with markers for specific compartments, such as MAP2 staining that is often used for dendritic analysis.

## Troubleshooting

### Problem 1: Cell density

Cell density is known to strongly influence the ability to correctly segment neuron soma and neurites in image-based analysis. When cell confluence is high or cells are clustered on the culture surface, soma and neurites overlap extensively and limit the ability to perform morphological analysis (step-by-step method details, step 2).

### Potential solution

• Low-density culture is broadly recommended for image-based neuron morphology analysis. Seed between 2,500 and 10,000 cells per well in a 96-well microplate, depending on the cell line used. Cell densities corresponding to ∼100 to 300 cells/mm^2^ will result in ∼50–150 cells analyzed per image at 20× magnification on the Agilent BioTek Cytation instrument.
• Optimize the SH-SY5Y differentiation protocol to limit cell proliferation and maintain cell numbers close to the initial seeding density.

### Problem 2: Cell distribution

SH-SY5Y cells, especially pathological models, tend to form aggregates, which complicates neurite and soma detection as described in problem 1 (step-by-step method details, step 3).

### Potential solution

• Select the most suitable surface coating to promote homogeneous cell distribution. Several coatings have been tested on SH-SY5Y cells (collagen, laminin, PLO/laminin), with laminin providing the most consistent results in our hands.
• After seeding, incubate the plate at 20°C for 1 h. Thermal gradients that form as the plate warms in the incubator can influence the positioning of cells that have not yet adhered to the surface, contributing to edge well effects and cell aggregation.

### Problem 3: Immunostaining and background signal

Immunostaining is critical to ensure accurate neurite detection by image analysis approaches. However, non-specific antibody binding can lead to high background fluorescence which interferes with image analysis (step-by-step method details, step 11).

### Potential solution

• Optimize primary and secondary antibody concentrations to avoid residual antibodies remaining in the wells.
• Ensure that blocking and washing steps are properly performed. Use BSA to block non-specific binding sites. Avoid washing immediately after the blocking step, as this may reduce blocking efficiency. Using an automated plate washer may help with washing consistency and reproducibility.

### Problem 4: Cell loss during washing steps

Washing steps are essential to minimize background signal (see problem 3), but excessive washing can lead to cell detachment from the well surface (step-by-step method details, step 9).

### Potential solution

• Choose a coating that ensures strong cell adhesion and minimizes detachment during washing.
• Perform washing steps manually using a multichannel pipette for greater control. For improved reproducibility, an automated microplate washer may also be used.

## Resource availability

### Lead contact

Further information and requests for resources and reagents should be directed to the lead contact, Dr. Amandine Grimm (amandine.grimm@unibas.ch).

### Technical contact

Technical questions regarding the execution of this protocol should be directed to the technical contact, Aline Broeglin (aline.broeglin@unibas.ch).

### Materials availability

This study did not generate new unique reagents.

### Data and code availability

• This study did not generate code.
• The datasets generated during this study are available on Open Science Framework: https://osf.io/26tgc/overview?view_only=1ca624c35de949a78bc3a8c9f8077813.
• This link provides the normalized data used for statistical analysis in GraphPad Prism. Data are organized by figure in separate sheets.

## Acknowledgments

This work was supported by grants from the OPO Foundation and the Dementia Research Synapsis Foundation Switzerland (2022-PI05). We are grateful to Fides Meier for the technical support.

## Author contributions

Performed the experiments, A.B. and A.G.; designed the experiments and analyzed the data, A.B., A.G., O.B., R.M., and A.R.; writing – original draft, A.B., A.G., O.B., and R.M.; funding acquisition, A.G.; resources, A.G. and A.E.; supervision, A.G. and A.E. All authors have read and agreed to the published version of the manuscript.

## Declaration of interests

R.M. and O.B. are employees and shareholders of Agilent Technologies, Inc.
